# Heritability Estimate for Antibody Response to Vaccination and Survival to a Newcastle Disease Infection of Native chicken in a Low-Input Production System

**DOI:** 10.3389/fgene.2021.666947

**Published:** 2021-09-30

**Authors:** Blaise Arnaud Hako Touko, Anold Tatah Kong Mbiydzenyuy, Tebug Thomas Tumasang, Julius Awah-Ndukum

**Affiliations:** ^1^Biotechnology and Bioinformatics Research Unit, Department of Animal Production, Faculty of Agronomy and Agricultural Sciences, University of Dschang, Dschang, Cameroon; ^2^Animal Research Lab, Department of Animal Sciences, School of Agriculture and Natural Resources, Catholic University Institute of Buea, Buea, Cameroon; ^3^Laboratory of Animal Physiology and Health, Department of Animal Sciences, University of Dschang, Dschang, Cameroon

**Keywords:** native chicken, heritability, resistance, selection, Newcastle disease

## Abstract

The Newcastle disease virus (NDV) is the deadliest chicken pathogen in low-input village poultry, and selecting for NDV resistance has been recommended as a sustainable strategy in backyard poultry production systems. However, selecting for disease resistance needs precision data from either a big population sample size or on many generations with good pedigree records for effective prediction of heritability (*h^2^*) and breeding values of the foundation stock. Such conditions are almost impossible to meet in low-input backyard production systems. This study aimed at proposing a realistic method for estimating the heritability of the immune response to vaccination and survival of NDV infection in village poultry production to inform a breeding strategy for ND resistance in Cameroon. A 1 and 3% selection intensity of cocks and hens for higher antibody (ab) response (ABR) to vaccination followed by progeny selection of chickens who survived an experimental NDV infection was conducted from an initial population of 1,702 chickens. The selection induced an increase of 1012.47units/ml (*p*<0.01) of the NDV antibody of the progeny as well as an effective survival rate (ESR) increase of 11.75%. Three methods were used to estimate the heritability ( $h^{2}$ ) of NDV antibody response to vaccination. $h^{2}$ was low irrespective of the method with estimates of 0.2227, 0.2442, and 0.2839 for the breeder’s equation method, the graphical method, and the full-sib/half-sib nested design, respectively. The mortality rate of infected chickens was high (86%). The antibody response to selection was not influenced by sex and genetic type even though the opposite was observed (*p*<0.05) for the ESR to NDV infection with naked neck chickens recording an ESR of 14% against 2.25% for the normal feather type. A very low heritability (0.0891) was observed for the survival against NDV infection. We confirm the evidence of disease resistance and the effect of selection for antibody response to vaccination on the improvement of the survival against NDV disease. Although the full sib/half sib nested design is more appropriate in case of availability of pedigree information, the direct methods are still useful in case of unavailability of full pedigree information. It is recommended that gene expression analysis should be prioritized for disease-resistance assessment and selection of native breeds of poultry.

## Introduction

In Cameroon, native local chickens contribute to poverty alleviation and food security of more than 60% of families living in rural areas (Fotsa et al., 2011). However, the breeding stock is almost always renewed every year due to high mortalities and poor management practices. Newcastle disease (ND) is the deadliest among others with 80% of birds in endemic areas in Tanzania (Buza and Mwamhehe, 2001), Southern Africa (Mtileni et al., 2012), and Cameroon (Hako Touko et al., 2015). Vaccination has proven not to be cost-effective in remote areas, and having genotypes that can resist or survive disease outbreaks is believed to be a sustainable strategy. Investigations have been carried out within native chickens of Cameroon, and some genetic markers associated with a high immune response to ND virus (NDV) infection have been identified (Hako Touko et al., 2015). There is a need to design and implement a breeding program in line with the valorization of ND resistance.

The estimation of heritability is a requirement for defining the appropriate improvement strategy. It is well documented that the heritability of antibody response (ABR) to the ND vaccine is low to moderate (Lwelamira et al., 2009; Liu et al., 2014; Rowland et al., 2018). However, that of ND resistance has not yet been estimated. It is paramount to determine the heritability of NDV resistance for local chickens of Cameroon to define the most appropriate strategy for its improvement. The estimation of genetic parameters is quite challenging in low-input poultry production considering the poor management conditions faced. Native chickens are reared as small-size flocks of 3–10 birds per household. They are unselected heterogeneous genetic resources randomly bred with little or no defined management system of feeding, housing, mating, or health management. There are no existing production records or pedigree information kept and no performance evaluation system. It is, therefore, currently impossible to have data on multiple generations for predictive analyses. These peculiarities make it difficult to obtain an initial and homogenous genetic resource for research and estimation of the genetic parameters.

In a previous study, a sample population was selected using LEI158 and MCW371 microsatellite markers of the major histocompatibility complex (MHC) B and quantitative trait loci (QTL) microsatellite alleles of favorable effect on antibody production against ND (Hako Touko et al., 2015). However, due to significant allele diversity, high selection intensity may be required for the improvement of antibody response and survival of NDV infection. This study aimed at proposing a realistic method for estimating the heritability of the immune response to vaccination and survival of NDV infection in village poultry production to inform a breeding strategy for ND resistance.

## Materials and Methods

The animal materials used in this research are not subject to any restriction and are approved by the scientific and ethical committee of the Department of Animal Science of the University of Dschang, No E0091/DZOO/FASA/UDs.

### Sampling and Experimental Procedure

The study was carried out at the experimental poultry farm of the School of Agriculture and Natural Resources of the Catholic University Institute of Buea, and serological tests were completed in the Laboratory of Animal Health of the Faculty of Agronomy and Agricultural Sciences of the University of Dschang. A total of 1,802 adult local chickens with no pedigree record were collected from farmers of the Community Based Management of Native Chickens Genetic Resources. The birds were 24weeks of age, including 912 normal feathered (610 hens and 302 cocks) and 890 naked necks (570 hens and 300 cocks). The birds were kept in the poultry farm for 28days for stabilization and acclimatization.

### G0, Gs, G1, and Control

After acclimatization, 1,000 eggs were collected from each parental stock (naked neck and normal feathered) and incubated to produce Parental; population (G0) day-old chicks for each group. G0 chicks were vaccinated against other viral diseases, including infectious bronchitis and Gumboro disease. Other breeding conditions, including prophylaxis, housing, and management were the same. Birds were fed *ad libitum*. Selected parents (Gs) consisted of two groups of equal size: The first group consisted of the top 1% of cocks and 3% of hens, including three cocks and 18 hens for the normal feathered and three cocks and 17 hens for the naked neck, selected for higher NDV antibody mean titer. This group was used to produced Offspring of selected population (G1; Gs offspring hatched from 900 fertile eggs, including 450 from normal feathered and 450 for naked neck). The second group was used for the NDV experimental infection of Gs. The control birds were considered for experimental infection and consisted of 50 normal feathered and 50 naked necks not vaccinated against NDV.

### Antibody Responsiveness to Vaccination

For antibody responsiveness to vaccination, G0, Gs, and G1 were tested at 28weeks of age. Blood samples were collected before vaccination against ND and 14days after vaccination. Sera from collected samples were isolated in 1.5-ml microtubes after sedimentation and screened for antibody titer. The vaccine used was Inmugal V.P. Hitchener B1 of Ovejero Laboratorios, Leon-Spain. An indirect ELISA method using Biochek Poultry Immunoassays for ND Antibody Test kit (catalogue code CK 116, www.asineh.com/PDF/Biochek; Product catalogue. PDF, pages 16–19) was used according to the strict instructions of the manufacturer. The relative amounts of antibodies in chicken samples were calculated by reference to the positive control and expressed as sample to positive (S/P) ratio and calculated as follows:

S/P=(Mean of Test Sample−Mean of negative control)/(Mean of Test sample-Mean of negative control)

For interpretation, samples with an S/P of 0.350 or greater contain anti-NDV antibodies and are considered positive. For the calculation of the titer, the following equation relates the S/P of a samples:

Log10 Titer=1.0 * Log(SP)+3.52, with antilog=Titer

### Experimental Infection

For the experimental infection, Gs and G1 were challenged at 7months of age. Experimental chicks were randomly sampled from 563 chicks, including 202 naked necks (T1) and 261 normal feathered chicks (T2). Four families of one cock and five hens were formed for each treatment, and 150 chicks were sampled per treatment, including 100 chicks of both sexes for T1 and 50 chicks for the first control (T1C) and the same for T2 and T2C. Experimental birds were reared identically and vaccinated against other common viral diseases, including Gumboro and infectious bronchitis. They were then challenged against the NDV, and records on survival were taken 14weeks after infection. All the birds that remained alive after the 14days of the trial were considered to have survived, and the survival rate (SR) was estimated. The experimental birds were then reared for 14 more days. Then, hens were mated with unchallenged healthy and fertile cocks and cocks with unchallenged healthy and fertile hens. After 2months, only hens that could lay and hatch fertile eggs were considered genetically viable, and the effective survival rate (ESR) of challenged birds was estimated.

### The APMV1 Strain

Isolates of Avian Paramyxovirus type 1 (APMV1) were harvested from postmortem diagnostics of sick chickens following the ND challenge. The NDV was cultured in fresh eggs and tested for pathogenicity index according to the standard operating manual in use. Randomly selected chicks were infected, and blood samples were collected before the infection and 14days after for antibody analysis. Then, viable birds capable of reproduction were presumed resistant and conserved for further studies.

### Statistical Analysis

Three methods, as detailed in Appendix 1–4, were used to estimate heritability, including the breeder’s equation method (Kelly, 2011), the graphical method adapted from Boyer (1958) and Verrier et al. (2009), and finally, the nested full-sib/half-sib analysis (Becker, 1975; Lynch and Walsh, 1998) and heritability method for discrete parameters (Razungles, 1977).

## Results

### Improvement for Antibody Responsiveness to Vaccination

The effect of selection for antibody responsiveness to vaccination on the antibody mean titer of the offspring according to genetic type and sex of the experimental birds (Tables 1 and 2) shows that all the birds tested negative for NDV antibody at 29weeks. For the same age, there was no significant difference between the genetic types and sex. The 1% selection intensity of cocks and 3% selection intensity of hens resulted in a significant (*p*<0.01) NDV antibody response of 1012.47units/ml corresponding to 16.77% ABR of the parental population from an average mean titer of 6,023 to 7,058units/ml for the offspring of selected parents as expected. However, the overall mean of antibody response to vaccination of selected parents (10,181units/ml; Table 2) was higher than that of their progeny at the same age (7,058units/ml).

**Table 1:** Antibody mean titer of parents, selected parents, and their offspring according to genetic types, sex, and age.

Genetic type	Sex (number of birds)	Age in Weeks	Antibody mean titrer (Unit/ml)			Sign
			Go	Gs	G1	
Naked neck	Cock (522)	29	314	311	300	ns^**^
		31	6083^c^	10,083^a^	7052^b^	
			(300)	(3)	(219)	
	Hen (818)	29	371	306	263	ns^**^
		31	6005^c^	10,205^a^	7089^b^	
			(570)	(17)	(231)	
Normal feathered	Cock (528)	29	298	315	304	ns^**^
		31	6109^c^	10,282^a^	6617^b^	
			(302)	(3)	(223)	
	Hen (855)	29	217	308	291	ns^**^
		31	5895^c^	10,129^a^	7055^b^	
			(610)	(18)	(227)	
Overall mean	(2723)	29	302	311	315	
		31	6036^c^	10,181^a^	7058^b^	
			(1782)	(41)	(900)	

a,b,c for the same age, between columns and for the same column between genetic types, variables bearing the same letters are statistically comparable (*p*>0.01).

ns, no significant difference observed;

G0, parental population;

Gs, selected parents;

G1, offspring from selected parents.

**Table 2:** Parameters of the distribution.

Parameters	Population mean $\left(\mu_{p}\right)$	Selected parents mean $\left(\mu_{s}\right)$	Mean offspring ( $\left(\mu_{p1}\right)$
Mean ( μ )	6036.01	10,181.95	7048.48
Standard Deviation $\left(\sigma \right)$	2391.04	1308.11	1029.94
Variance $\left(\sigma^{2}\right)$	5,717,105.40	1,711,155.38	1,128,366.22
Coefficient of variation	39.61	12.85	14.61

### Heritability Estimate of the Antibody Responsiveness to Vaccination

The analysis of the parameters of distributions (Table 3) shows that the 1% sire and 3% dam selection decreased the SD with a coefficient of variation of 13 and 15%, respectively, for selected parents and their offspring as compared with 39.61% for the population. *h^2^* was moderate irrespective of the method with estimates of 0.2227, 0.2442, and 0.2839 for the breeder’s equation and the graphical methods (Table 4) and the full-sib/half-sib nested design (Table 5), respectively.

**Table 3:** Selection parameters and heritability estimate from the direct method.

Selection parameters	Formula	Estimate
$\Delta_{ps}$	$\mu_{s}-\mu_{p}$	4145.94
$\Delta_{p1}$	$\mu_{p1}-\mu_{P}$	1012.47
$h_{1}^{2}$	$\Delta_{p1}/\Delta_{ps}$	0.2227
$h_{2}^{2}$	$\frac{P_{offspring}-{\bar{P}}_{population}}{\left(P_{selectedparents}-{\bar{P}}_{population}\right)}$	0.2442

$$\Delta_{ps}=selectiondifferential.$$

$$\Delta_{p1}=G1\;superiority=geneticgain.$$

$$h_{1}^{2}=heritabilityfromthegraphicalmethod.$$

$$h_{2}^{2}=heritabilityfromthebreeder^{\prime }sequation.$$

**Table 4:** Heritability estimate from variance component.

Factor	df	MS	E(MS)	$\sigma^{2}$	$h^{2}$
Sires	5	13,318,469.9	$\sigma_{e}^{2}$ +10 $\sigma_{d}^{2}+60\sigma_{s}^{2}$	76,057.5362	0.2839
Dam/Sire	30	3,839,841.5	$\sigma_{e}^{2}$ +10 $\sigma_{d}^{2}$	−75,038.6533	
Sibb/Dam	324	996,554.475	$\sigma_{e}^{2}$	1,070,373.33	

**Table 5:** ANOVA of the survival to Newcastle disease virus (NDV) infection.

Factor	df	SS	MS	E(MS)	σ	$h^{2}$
Sires	3	0,36	0,1200	$\sigma_{w}^{2}+50\sigma_{s}^{2}$	$\sigma_{s}^{2}=-0,0077$	$\frac{4\sigma_{s}^{2}}{\sigma_{w}^{2}+\sigma_{d}^{2}+\sigma_{s}^{2}}$ =0.0891
Dam/Sire	196	−36,04	−0,1839	$\sigma_{w}^{2}+0,8163\sigma_{d}^{2}$	$\sigma_{d}^{2}=-0,8459$	
Sibb/Dam	150	76	0,5066	$\sigma_{w}^{2}=0,5066$	$\sigma_{w}^{2}=0,5066$	
Total	199	40,32	0,2026			

### Heritability Estimate of Survival of NDV Infection

The estimate of the heritability of survival of NDV infection (Table 5) is very low=0.0891, showing that only 9% of the superiority of selected resistant parents is transferred to their immediate generation.

### Survival of NDV Infection

For the effect of the selection for antibody responsiveness to ND vaccination on the survival of ND infection of the progeny of local chickens (Table 6), the selection intensity of 1% cocks and 3% hens has led to an improvement of the survival rate from 6 to 21%, equivalent to a mortality rate of 94 to 79% and of the ESR from 2.25 to 14%. The improvement of the survival against ND infection is observed for all experimental groups irrespective of the genetic type or sex.

**Table 6:** Effect of the selection of parent for antibody responsiveness to vaccination on the survival of local chicken according to genetic group and sex.

Genetic type	Sex	Parents			Offspring		
		Number of birds	Survival (%)	Effective survival (%)	Number of birds	Survival (%)	Effective survival (%)
Naked Neck	Cock	50	8	2	50	21	14
	Hen	50	4	3	50	17	14
Normal feathered	Cock	50	2	0	50	16	11
	Hen	50	6	4	50	21	17
Overall		200	5	2.25	200	18.75	14

## Discussion

The progeny of immunized parents were all NDV-ab negative at 29weeks. It was previously confirmed that parental NDV-ab of immunized birds is present in the blood and effective to protect their chicks only 10days post-hatchery (Hako Touko et al., 2015). Similar findings of Shahid and Liaquat (2017) reported that maternal NDV-ab protects their progeny during the 1st week of life. The selection for high parental NDV-ab response improved NDV-ab response of their sibs 16.77% and survival of infection 15% SR and 11.75% ESR, respectively. ESR is more appropriate to conclude about the effectiveness of the selection as only birds that survive the infection and are capable of reproducing are considered genetically viable.

The significant increase (*p*<0.01) of antibody titer (1012.47units/ml) of the offspring as compared with the population mean induced a correlated response of 11.75% ESR of offspring of selected parents. This is evidence that the selection for antibody responsiveness to vaccination against NDV improves natural immunity against NDV and can, therefore, be recommended for selection to improve disease tolerance or resistance. Antibody response plays an important role in host resistance to ND, and selection for antibody response can effectively improve disease resistance in chickens (Luo et al., 2013). Moreover, chickens with high NDV-ab titers were associated with a higher frequency of MHC-B and QTL alleles of favorable effect on disease resistance in chickens (Hako Touko et al., 2015). Despite the improvement of the immune response in the progeny, the mortality rate observed in this study remains high (86%). A lower rate (83.72%) was earlier reported in the field (Hako Touko et al., 2015) although higher mortalities (90–100%) were recorded by several authors (Nanthakumar et al., 2000; Orsi et al., 2010; Spickler, 2016). This justifies why NDV is included in “listed” agents or reportable diseases by the Office International des Epizooties (OIE; Aldous and Alexander, 2001; Boynukara et al., 2013) and is considered to be the number one disease constraint of economical importance in poultry production around the globe with more impact in low- and middle-income countries (Liu et al., 2014; Deist et al., 2017; Rowland et al., 2018). The high mortality rate from experimental infection of unvaccinated birds is a call for concern, and the method of selection from experimental infections should be restricted.

The heritability of NDV-ab response to vaccination was estimated from the Breeder’s equation (Kelly, 2011), graphical method (Verrier et al., 2009), and ANOVA full-sib/half-sib nested design (Lynch and Walsh, 1998) and were all low (0.2227, 0.2442, and 0.2839). It is known that low heritability is an indication that the genetic gain from the mass selection is very low. Consequently, the improvement of the antibody responsiveness to vaccination against NDV through selection is time-consuming. In this situation, an improvement of environmental factors and management practices, including feeding, housing, and vaccination, is likely to minimize the incidence of NDV infection and improve poultry productivity in low- and middle-income countries. All three estimates are less than the heritability estimates of 0.29, 0.31, and 0.48 earlier reported in low-input production systems (Peleg et al., 1976; Liu et al., 2014; Lwelamira, 2012). Statistically, the range of the heritability estimate is [0,1].

The heritability obtained for the survival to infection was lower (0.0891) compared with that of antibody response to the vaccination (0.2442). Considering the selection, lower values of heritability would be expected from the unselected population of indigenous chickens of Cameroon. These observations expose that parental improvement of low heritability traits such as survival of infection due to the selection for high antibody response is low. This may be explained by the fact that survival of infection as disease resistance is a complex trait under multiple genes’ influence with each gene inducing only part of the overall effect (Fulton et al., 2006). In this context, bigger population size may better reflect the polygenetic mode of action of expected genes. The low ESR, despite the moderate antibody response to vaccination, may also be correlated with the velogenic nature of the NDV (Shabbir et al., 2012).

In this study, the higher survival or lower mortality rate and higher antibody titer are indicators of improved resistance to NDV infection. Previous studies define resistance as the ability of the host to interfere with the pathogen life cycle in various ways, including lower pathogen load, higher antibody, and less morbidity and/mortality (Rauw, 2012; Bishop, 2014; Deist et al., 2017). We used two variables as indicators of survival, including SR and ESR. The second that is either equal to or lower than the first gives the percentage of individuals that survive the infection and are capable of reproduction. Therefore, ESR is more reliable to conclude about the disease-resistance status of a breed as well as for the estimation of genetic parameters. Despite the selection for high antibody response, the level of resistance improvement was still low as evidenced by the relatively low SR obtained. It is known that the immune response to viruses is very complex, and antibody response to NDV is a quantitative trait under polygenic control (Yonash et al., 2001; Biscarini et al., 2010). According to Liu et al. (2014), many significant markers influence the innate and adaptative immune response of chickens, but none of them can explain more than 5% of the phenotypic variance.

## Conclusion

The 1% selection intensity of cocks and 3% selection intensity of hens resulted in a significant increase (*p*<0.01) of the NDV antibody response of 1012.47units/ml corresponding to 16.77% ABR of the parental population from an average mean title of 6,023 to 7,058units/ml for the offspring of selected parents. Low values of heritability for antibody responsiveness and survival to experimental infection confirm that mass selection is not effective for the improvement of natural resistance against NDV. Disease resistance being a quantitative trait under polygenic control, it is, therefore, suggested that a genome-wide associated study be conducted in view of identifying more genes involved and proposing an efficient selection strategy.

## Data Availability Statement

The original contributions presented in the study are included in the article/Supplementary Material, further inquiries can be directed to the corresponding author.

## Ethics Statement

The animal study was reviewed and approved by the Ethics and Scientific Committee of University of Dschang.

## Author Contributions

BH designed the study, performed statistical analysis of the data, field supervision of the project, and drafting of the manuscript, and wrote the manuscript. AK managed experimental birds and collected data. TT carried out the laboratory test. JA-N provided the general supervision of the work. All authors contributed to the article and approved the submitted version.

## Funding

The study was funded by IFS/COMSTECH Research grant Upgrading natural disease resistance against Avian paramyxovirus 1 (Newcastle disease) and *in situ in vivo* conservation of selected local chickens Grant B/5115-2.

## Conflict of Interest

The authors declare that the research was conducted in the absence of any commercial or financial relationships that could be construed as a potential conflict of interest.

## Publisher’s Note

All claims expressed in this article are solely those of the authors and do not necessarily represent those of their affiliated organizations, or those of the publisher, the editors and the reviewers. Any product that may be evaluated in this article, or claim that may be made by its manufacturer, is not guaranteed or endorsed by the publisher.

## Abbreviations:

ab: Antibody
ABR: Antibody response
APMV1: Avian Paramyxovirus type 1
CBM: Community-based management
ESR: Effective survival rate
GWAS: Genome wide association studies
G0: Parental; population
G1: Offspring of selected population
Gs: Selected parents
*h^2^*: Heritability
MHC: Major histocompatibility complex
Na: Naked neck gene
ND: Newcastle disease
NDV: Newcastle disease virus
QTL: Quantitative trait loci
SNP: Single nucleotide polymorphism
TSR: Total survival rate.
