# Supplementary material for: Heritability Estimate for Antibody Response to Vaccination and Survival to a Newcastle Disease Infection of Native chicken in a Low-Input Production System
Source: Front Genet. 2021 Sep 30;12:666947. doi: 10.3389/fgene.2021.666947 (PMC8514834; doi:10.3389/fgene.2021.666947)
Supplement: Supplementary file 1 [file Data_Sheet_1.DOCX]

**Additional file 1** Estimation heritability adapted from Boyer et al (1958)

The genotype value $(g)$ is estimated as follow:

$g_{population}=P_{population}-E_{population}$ (1)

$g_{selected parents}=P_{selected parents}-E_{selected parents}$(2)

$g_{offspring from selection}=P_{offspring from selection}-E_{offspring from selection}$ (3)

$(g_{offspring from selection}-\bar{g}_{population})=h^{2}(P_{selected parents}-\bar{P}_{population})$ (4)

Assuming that the environmental factor is controlled by the experimental design, the following equation can be deducted from (1), (2) and (3):

$E_{population}=E_{selected parents}=E_{offspring from selection}=E$(5)

(5)$\leftrightarrow$(4)=$(P_{offspring from selection}-E- \bar{P}_{population}+E)=h^{2}(P_{selected parents}-\bar{P}_{population})$

$\leftrightarrow(P_{offspring from selection}- \bar{P}_{population})=h^{2}(P_{selected parents}-\bar{P}_{population})$

thus ,

$h^{2}=\frac{(P_{offspring from selection}- \bar{P}_{population})}{(P_{selected parents}-\bar{P}_{population})}$
