# Supplementary material for: Heritability Estimate for Antibody Response to Vaccination and Survival to a Newcastle Disease Infection of Native chicken in a Low-Input Production System
Source: Front Genet. 2021 Sep 30;12:666947. doi: 10.3389/fgene.2021.666947 (PMC8514834; doi:10.3389/fgene.2021.666947)
Supplement: Supplementary file 2 [file Data_Sheet_2.DOCX]

**Additional file 2** The heritability estimation as adapted from Verrier et al (2009)

$y_{i}\sim$N ($\mu_{p}, \sigma^{2})$ being a formulation of the normal distribution (figure 1) of antibody responsiveness to vaccination of the experimental birds where

- $y_{i}$ is the antibody mean titre;
- $\mu_{p}$ the mean of the population;
- $\sigma^{2}$ the variance of the population.


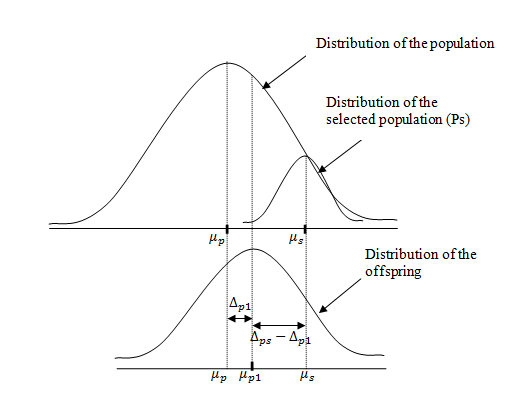


Normal distribution graph of experimental populations

The heritability ($h^{2})$ of the selective superiority for antibody responsiveness to vaccination against NDV can be defined as proportion of the superiority of the selected population that is transmitted to the offspring with:

$\Delta_{ps}$= $\mu_{s}-\mu_{p}$ the estimate of the superiority of the selected birds above the overall population;

$\Delta_{p1}{=\mu}_{p1}-\mu_{P}$ the estimate of the superiority of the offspring $p_{1}$above the overall population

$\Delta_{p1}$= the proportion of $\Delta_{ps}$ transmitted to the offspring $p_{1}$

$$h^{2}=\Delta_{p1}/ \Delta_{ps}$$
