# Supplementary material for: Heritability Estimate for Antibody Response to Vaccination and Survival to a Newcastle Disease Infection of Native chicken in a Low-Input Production System
Source: Front Genet. 2021 Sep 30;12:666947. doi: 10.3389/fgene.2021.666947 (PMC8514834; doi:10.3389/fgene.2021.666947)
Supplement: Supplementary file 3 [file Data_Sheet_3.DOCX]

**Additional file 3** Estimation of heritability following the Nested full-sib/half-sib analysis (Becker, 1975).

The statistical model for the nested design was defined as

$$Y_{ijk}=\mu+s_{i}+d_{ij}+e_{ijk}$$

where $Y_{ijk}$ is the phenotype of the $k^{th}$offspring from the family of the $i^{th}$ sire and $j^{th}$ dam, $s_{i}$ is the effect of the $i^{th}$ sire, $d_{ij}$ is the effect of the $j^{th}$ dam mated to the $i^{th}$ sire, and $e_{ijk}$is the residual uncontrolled environmental and genetic deviation (the within-full-sib family deviations). It is assumed that all effects are random, normal and independent with expectations equal to zero. The components of variances and the heritability estimates were obtained according to Becker (1975). The ANOVA table is presented in table 1.

ANOVA for the antibody responsiveness to vaccination

| Factor | degree of freedom | Sum of Squares  (SS) | Mean square  (MS) | E(MS) |
| --- | --- | --- | --- | --- |
| Sires | $S-1$ | nd $\sum_{i=1}^{s} \sum_{j=1}^{d} (\mu_{i}-{\mu)}^{2}$ | ${SS}_{s}/S-1$ | $\sigma_{e}^{2}+n\sigma_{d}^{2}+nd\sigma_{s}^{2}$ |
| Dam/Sire | $S(D-1)$ | n $\sum_{i=1}^{s} \sum_{j=1}^{d} (\mu_{ij}-{\mu_{i})}^{2}$ | ${SS}_{d}/S(D-1)$ | $\sigma_{e}^{2}+n\sigma_{d}^{2}$ |
| Sibb/Dam | $SD(N-1)$ | $\sum_{i=1}^{s} \sum_{j=1}^{d} \sum_{k=1}^{n} {(Y}_{ijk}-{\mu_{ij})}^{2}$ | ${SS}_{e}/SD(N-1)$ | $\sigma_{e}^{2}$ |

$$\sigma_{e}^{2}= {MS}_{e}$$

$$\sigma_{d}^{2}= \frac{({MS}_{d}-{MS}_{e})}{n}$$

$$\sigma_{s}^{2}= \frac{({MS}_{s}-{MS}_{d})}{nd}$$

$$h^{2}=\frac{4\sigma_{s}^{2}}{\sigma_{e}^{2}+\sigma_{d}^{2}{+\sigma}_{s}^{2}}$$
