# Supplementary material for: Heritability Estimate for Antibody Response to Vaccination and Survival to a Newcastle Disease Infection of Native chicken in a Low-Input Production System
Source: Front Genet. 2021 Sep 30;12:666947. doi: 10.3389/fgene.2021.666947 (PMC8514834; doi:10.3389/fgene.2021.666947)
Supplement: Supplementary file 4 [file Data_Sheet_4.DOCX]

**Additional File 4** Heritability of resistance against NDV infection (Razungles, 1977)

ANOVA of resistance to NDV disease infection.

| Factor | Degree of freedom | Sum of  square | Mean square | E(MS) | $\sigma$ |
| --- | --- | --- | --- | --- | --- |
| Sires | $d_{1}= S-1$ | $SS-{SS}_{s}$ | ${SS}_{1}/d_{1}$ | $\sigma_{w}^{2}+k_{1}\sigma_{d}^{2}+k_{2}\sigma_{s}^{2}$ | $\sigma_{s}^{2}=\frac{{(MS}_{1}-{MS}_{2})-\sigma_{d}^{2}(k_{1}-k_{3})}{k_{2}}$ |
| Dam/  Sire | $d_{2}=\sum_{i=1}^{s} (N_{i}-1)$ | ${SS}_{s}-{SS}_{d}$ | ${SS}_{2}/d_{2}$ | $\sigma_{w}^{2}+k_{3}\sigma_{d}^{2}$ | $\sigma_{d}^{2}=\frac{{MS}_{2}-{MS}_{3}}{k_{3}}$ |
| Sibb/  Dam | $d_{3}=N-\sum_{i=1}^{s} N_{i}$ | ${SS}_{d}-{SS}_{e}$ | ${SS}_{3}/d_{3}$ | $\sigma_{w}^{2}$ | $\sigma_{w}^{2}$ |
| Total | $d_{4}= N-1$ | $SS=\frac{a(n-a)}{n}$ |  |  |  |

$$k_{1}= \sum_{i} (\sum_{ij} n_{ij}^{2}/N_{i})-\sum_{ij} n_{ij}^{2}/N)/ (S-1)$$

$$k_{2}=(N-\sum_{i} N_{i}^{2}/N) / (S-1)$$

$$k_{3}=N-\sum_{i} (\sum_{j} n_{ij}^{2}/N_{i})/\sum_{i} \left( N_{i}-1 \right)$$

$SS=\frac{a\left( n-a \right)}{n}$ , $a being the total number of survivors and n the total number of$

$$individuals.$$

${SS}_{1}= SS-{SS}_{s}$ ; ${SS}_{s} being the sum of "\text{s" }terms of \frac{a\left( n-a \right)}{n}, each being estimated$

$$seperatelly for each family of sire.$$

${SS}_{2}= {SS}_{s}-{SS}_{d}$ ; ${SS}_{d} being the sum of \sum_{i=1}^{s} N_{i}\text{ }terms of \frac{a\left( n-a \right)}{n}, each being$

$$estimated seperatelly for each family of dam.$$

${SS}_{3}= {SS}_{d}-{SS}_{w}$ ; ${SS}_{w}being the sum of \sum_{ij} n_{ij}terms of$ $\frac{a\left( n-a \right)}{n}.$
